# A Case Report of Extrapulmonary Tuberculosis: A Potpourri of Imaging Manifestations

**DOI:** 10.7759/cureus.114381

**Published:** 2026-08-11

**Authors:** Rohit Chakravarty, Hirdesh Sahni, Nilanjan Sarkar

**Affiliations:** 1 Radiology, Tata Main Hospital, Jamshedpur, IND

**Keywords:** extrapulmonary tuberculosis, femoral neck abscess, obturator internus abscess, retroperitoneal lymphadenopathy, ureteric stricture

## Abstract

Extrapulmonary tuberculosis (EPTB) can mimic pyogenic infection, inflammatory disease, and malignancy. We report the case of a middle-aged female patient who presented with low-grade fever, left hip pain, and left loin pain. Pelvic imaging demonstrated osteomyelitis with an abscess centered in the left femoral neck. An abscess was also present in the left obturator internus muscle. Further abdominal imaging demonstrated left hydronephrosis with a mid-ureteric stricture and retroperitoneal lymphadenopathy. Pus was aspirated and tested positive for *Mycobacterium tuberculosis*, establishing the diagnosis of tuberculous osteomyelitis with abscess formation and presumptive multifocal extrapulmonary involvement, supported by imaging findings and interval regression. Antitubercular therapy was initiated, and ureteric stenting was performed to relieve the symptoms of urinary obstruction. Follow-up imaging after completion of treatment showed complete resolution of the disease. This case highlights the complementary role of imaging in mapping the multisystem disease burden, guiding microbiological confirmation, and documenting the treatment response.

## Introduction

Tuberculosis remains a major global infectious disease, and extrapulmonary tuberculosis (EPTB) continues to pose a substantial diagnostic challenge because its clinical manifestations are often nonspecific and its imaging appearances can be highly variable [1]. For radiologists, EPTB is particularly important because it frequently presents as a multisystem process that mimics pyogenic infection, inflammatory disease, or malignancy [1].

Musculoskeletal tuberculosis is an established form of EPTB, but extraspinal involvement may be uncommon, deceptively indolent, and associated with delayed diagnosis [2-4]. Skeletal muscle tuberculosis is rare, likely because skeletal muscle provides an unfavorable milieu for mycobacterial proliferation, and is usually recognized only when it manifests as abscess formation or contiguous spread from adjacent disease [4,5]. Genitourinary tuberculosis likewise has protean manifestations and may involve the kidneys, ureters, bladder, or genital tract, with ureteric inflammation and stricture causing obstructive uropathy when the diagnosis is delayed [6-9].

We report a microbiologically confirmed case of tuberculous osteomyelitis with abscess formation and presumed multifocal involvement in a 40-year-old woman. There was simultaneous involvement of the left femoral neck/proximal femur, left obturator internus muscle, retroperitoneal lymph nodes, and left ureter. The case is notable for its broad radiologic spectrum and the way in which multimodality imaging helped unify apparently unrelated abnormalities into a single diagnosis.

## Case presentation

**Figure 1:**
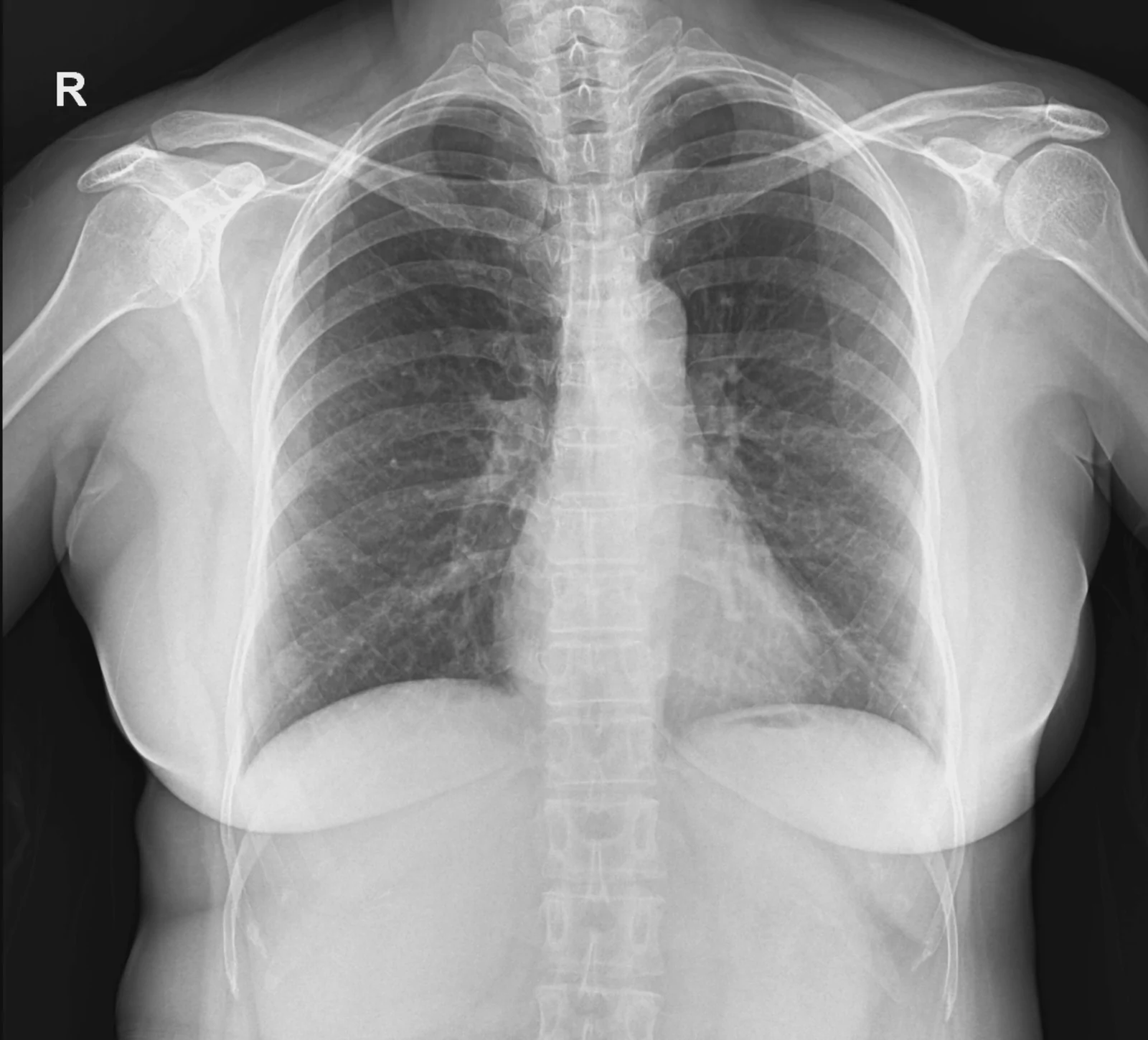
Posteroanterior chest X-ray showing no obvious abnormality. The patient’s chest X-ray shows no evidence of focal consolidation, pleural thickening, or pleural effusion. No radiographic features suggestive of pulmonary tuberculosis are seen.

A 40-year-old woman presented with low-grade fever and left hip pain for one month, along with left loin pain for 15 days. She had no history of trauma, prior surgery, autoimmune disease, or other relevant comorbidities. HIV serology was negative. On examination, she had an average build, a temperature of 100°F, stable vital signs, a limping gait, and deep left hip pain that increased markedly with movement. Laboratory evaluation demonstrated leukocytosis, neutrophilia, elevated CRP, and an elevated ESR, favoring an infectious etiology (Table 1). Her serum creatinine level was elevated (1.6 mg/dL). The chest X-ray was normal (Figure 1); hence, sputum culture was not performed.

**Table 1:** Laboratory findings showing abnormal results and corresponding normal reference ranges. The table shows leukocytosis and neutrophilia, with elevated ESR, CRP, and serum creatinine levels. These findings suggest an infectious etiology. Normal reference ranges for the laboratory parameters are provided for comparison [10].

Laboratory parameter	Result	Normal reference range
Total leukocyte count	17,000 cells/µL	4,000-11,000 cells/µL
Absolute neutrophil count	8,900 cells/µL	1,500-7,500 cells/µL
ESR	35 mm/h	Men: 0-15 mm/h; women: 0-20 mm/h
CRP	18 mg/L	<5 mg/L
Serum creatinine	1.6 mg/dL	Men: 0.74-1.35 mg/dL; women: 0.59-1.04 mg/dL

**Figure 2:**
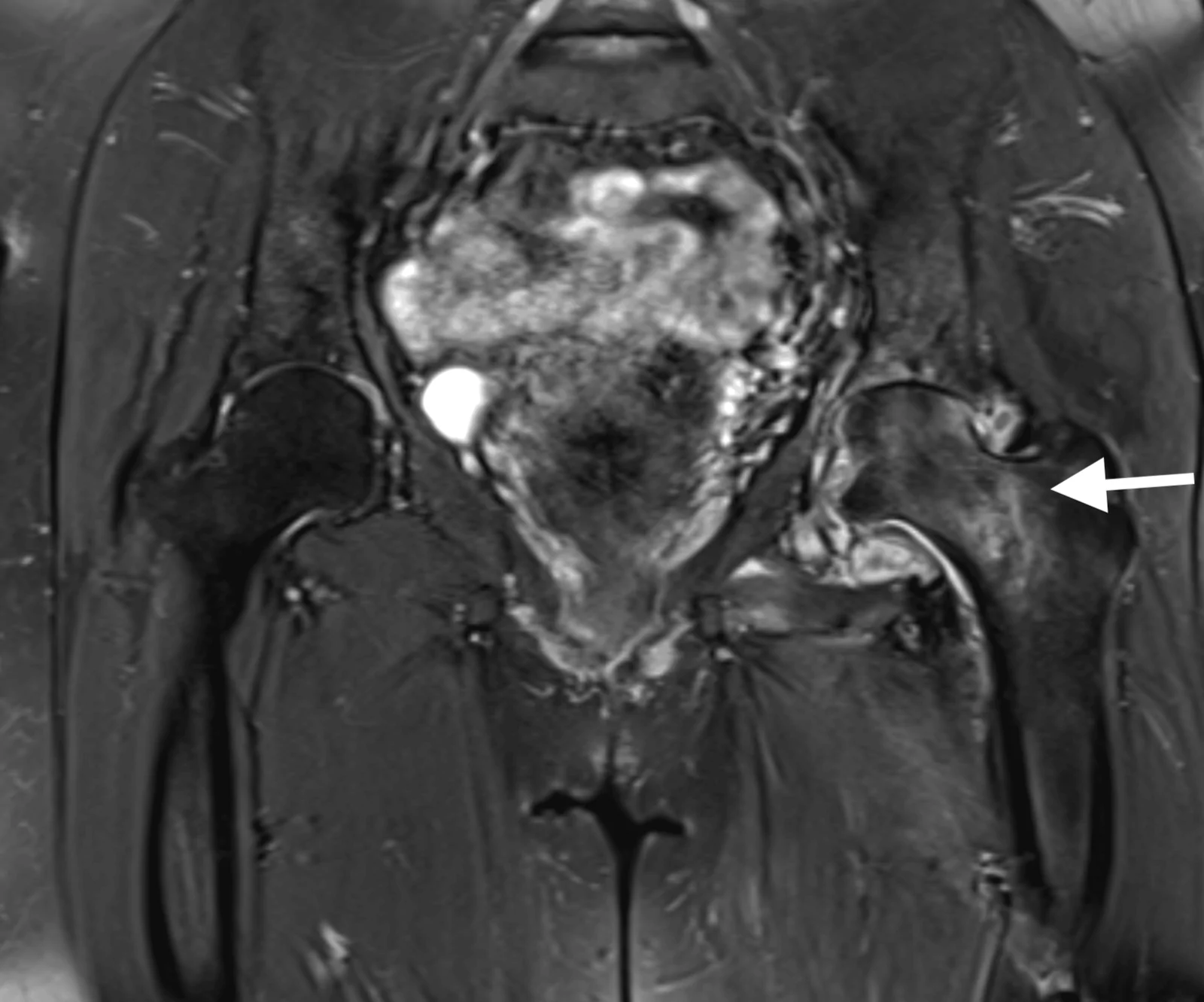
Coronal fat-suppressed T2-weighted MRI of the pelvis showing left femoral osteomyelitis with intraosseous abscess formation (arrow) and joint effusion. The image shows a hyperintense area in the left femoral neck corresponding to osteomyelitis with intraosseous abscess formation, as indicated by the arrow. The abscess measures 3.5 × 2.2 × 1.3 cm in the craniocaudal, transverse, and anteroposterior dimensions, respectively. A reactive joint effusion is also present.

**Figure 3:**
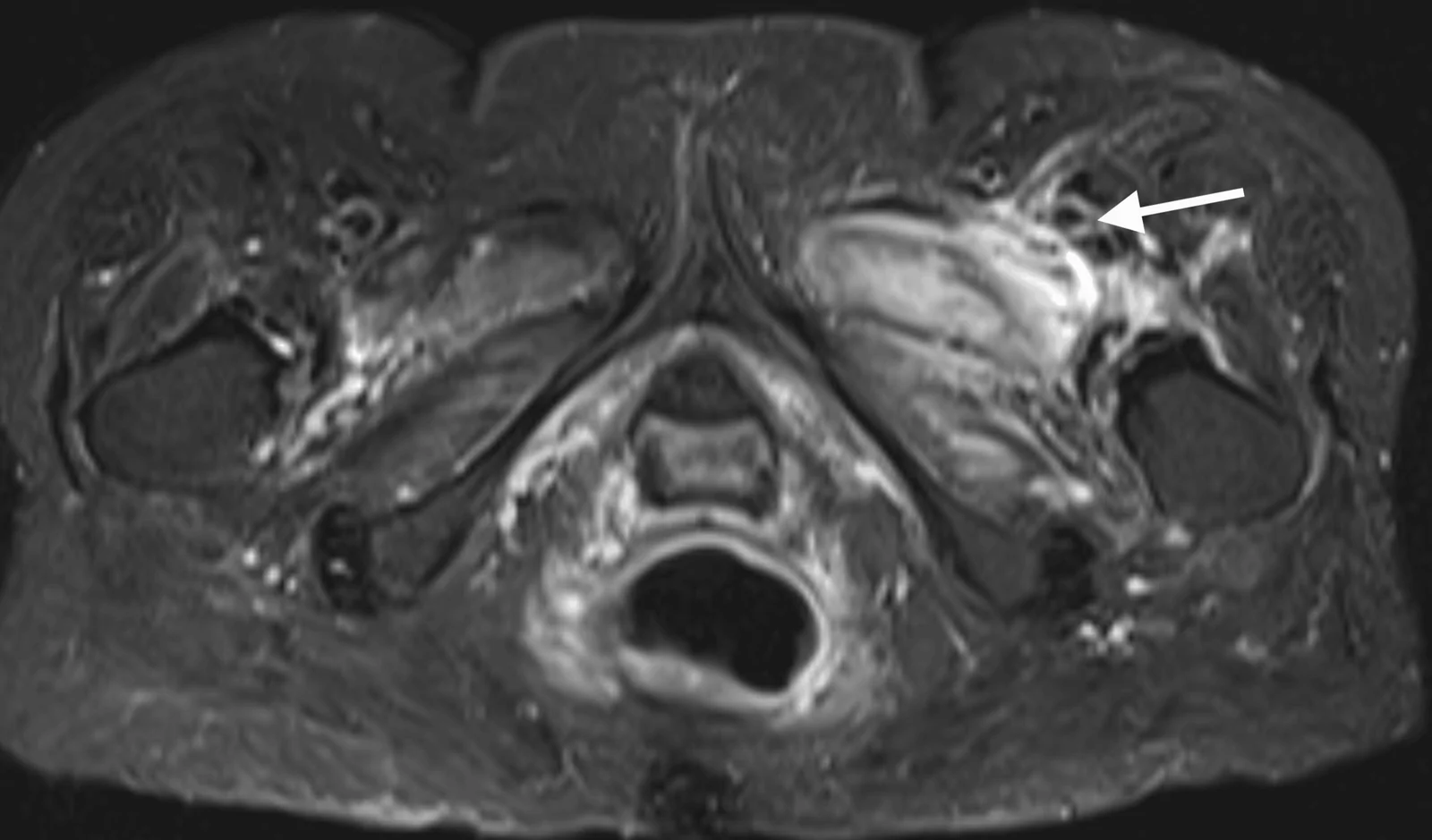
Axial fat-suppressed T2-weighted MRI of the pelvis showing left obturator internus myositis with abscess formation (arrow). The image shows hyperintense signal and enlargement of the left obturator internus muscle, with inflammatory changes in the adjacent fat, as indicated by the arrow. These findings are consistent with myositis with abscess formation.

MRI of the pelvis revealed an abscess involving the left femoral neck with surrounding marrow edema (Figure 2). An associated abscess was also identified in the left obturator internus muscle (Figure 3).

**Figure 4:**
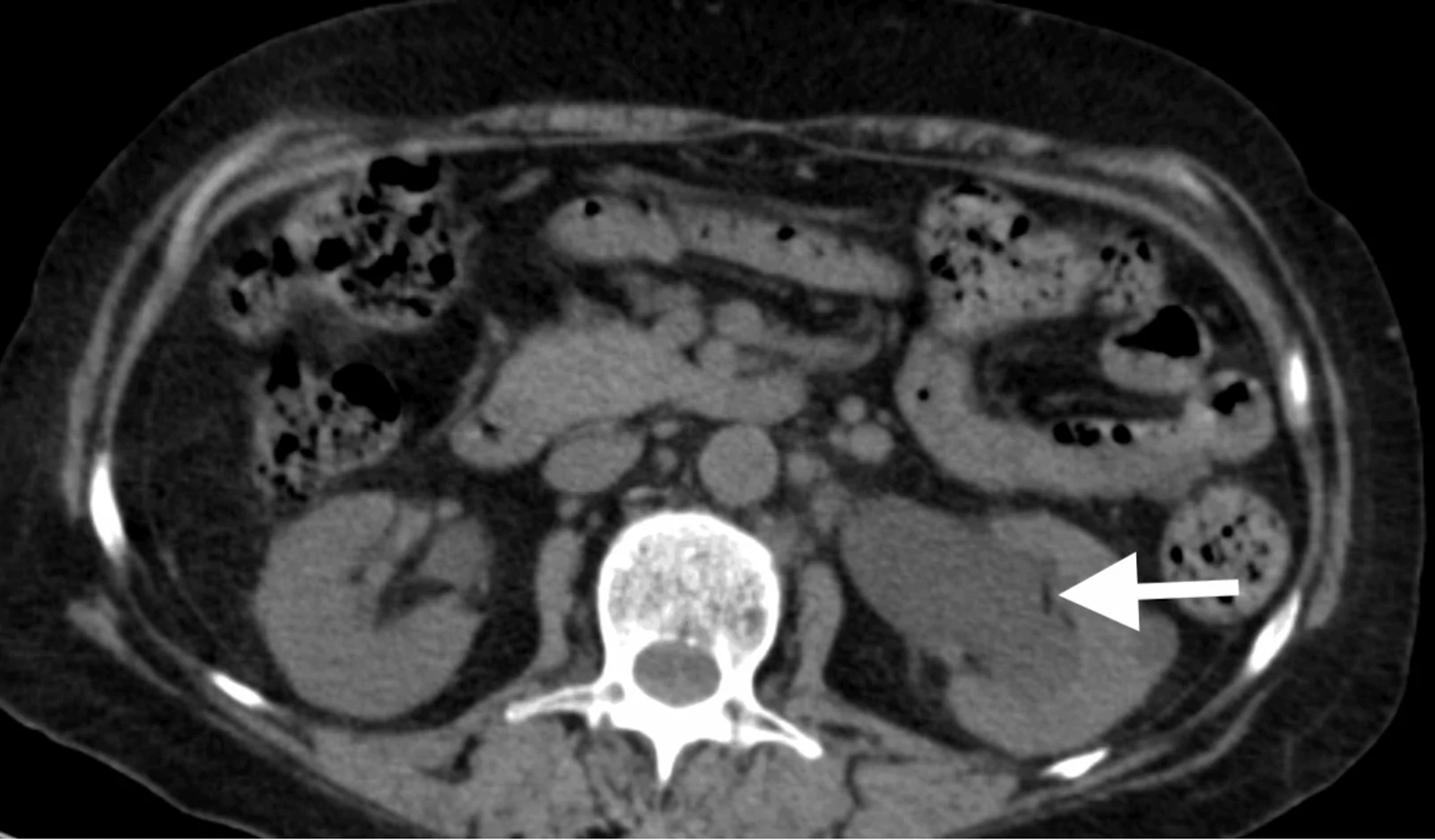
Axial non-contrast CT image of the abdomen showing left hydronephrosis (arrow). The image shows a grossly dilated left renal pelvis, as indicated by the arrow. The renal parenchyma appears normal in morphology and attenuation.

**Figure 5:**
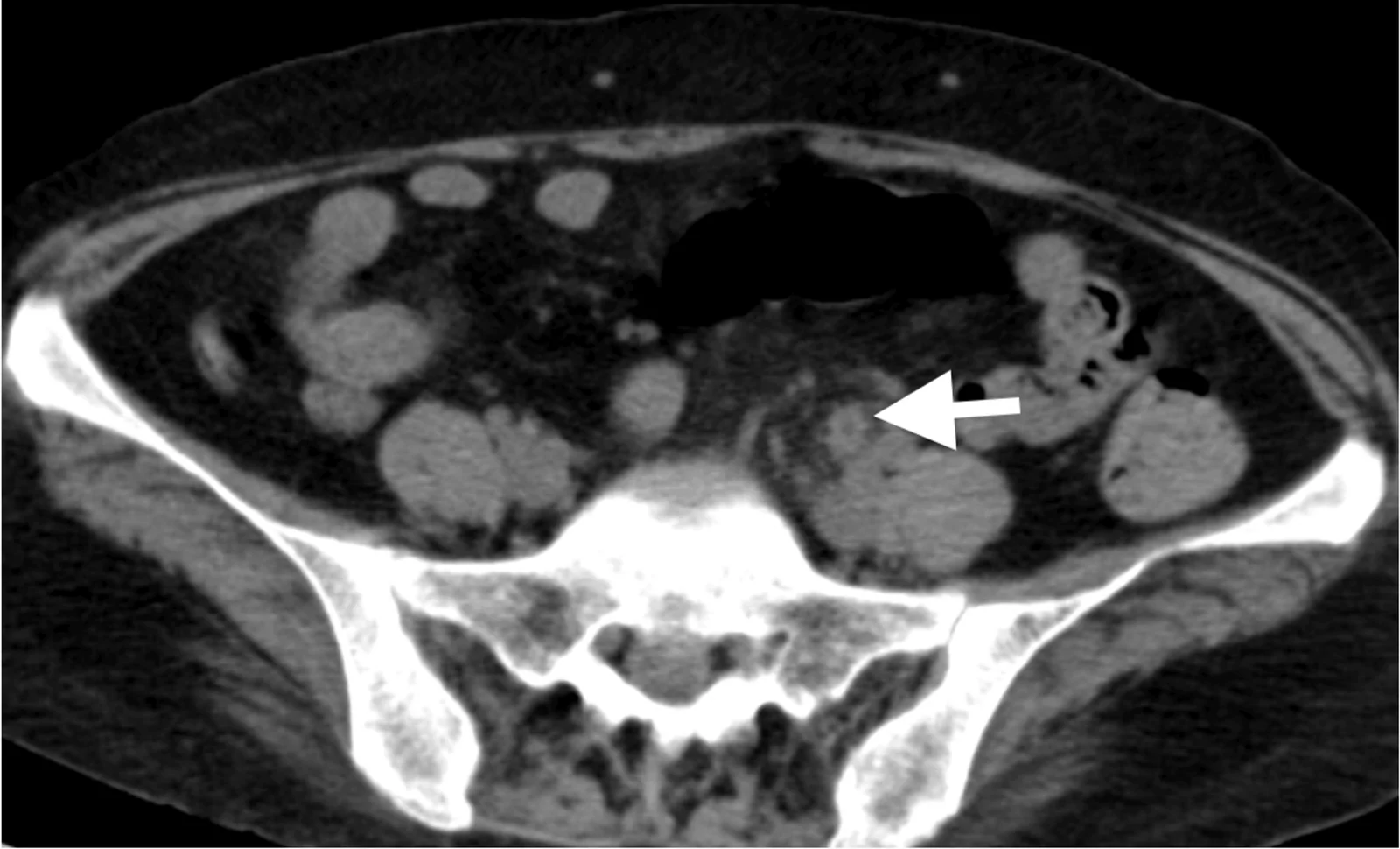
Axial non-contrast CT image of the abdomen showing a left ureteric stricture with adjacent fat stranding suggestive of inflammation (arrow). The image shows thickening of the left ureteric wall with partial luminal narrowing and adjacent periureteric fat stranding, suggesting the possibility of an inflammatory left ureteric stricture. The strictured segment measures 1.2 cm in craniocaudal length and is located at the level of the S1 vertebra. No ureteric mass lesion or calculus is seen.

**Figure 6:**
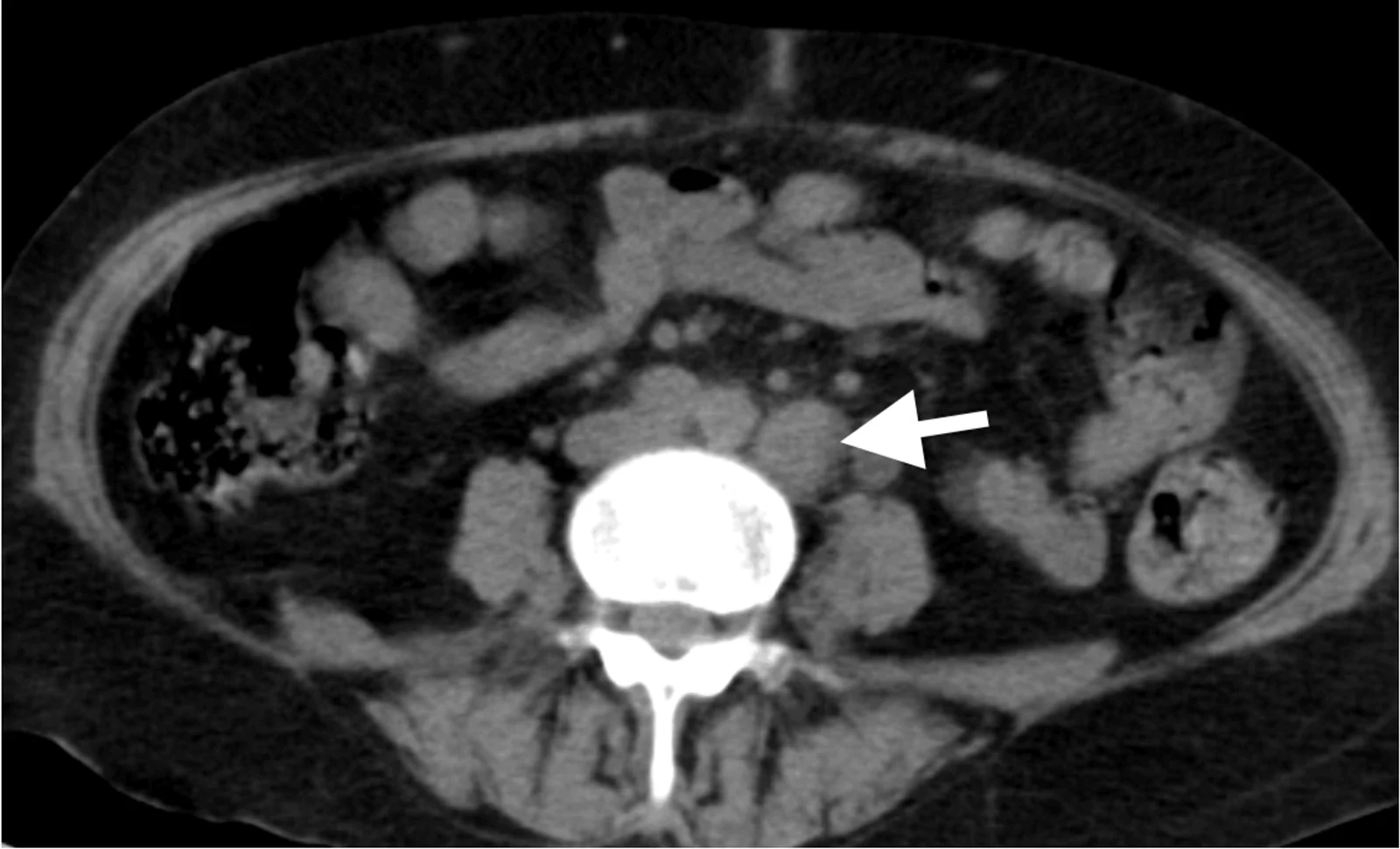
Axial non-contrast CT image of the abdomen showing multiple enlarged retroperitoneal lymph nodes in the preaortic and para-aortic regions (arrow). The image shows multiple enlarged retroperitoneal lymph nodes in the preaortic and para-aortic regions, as indicated by the arrow. In the appropriate clinical context, these findings may be related to an infectious etiology. The largest lymph node measures approximately 1.8 cm in short-axis diameter.

Subsequent non-contrast CT of the abdomen was performed because the serum creatinine level was elevated. It demonstrated left hydronephrosis (Figure 4) with an inflammatory mid-ureteric stricture (Figure 5). Multiple enlarged retroperitoneal lymph nodes were also observed (Figure 6). The coexistence of musculoskeletal disease, nodal enlargement, and genitourinary obstruction broadened the differential diagnosis to include tuberculosis, disseminated bacterial infection, lymphoma, and metastatic disease.

**Figure 7:**
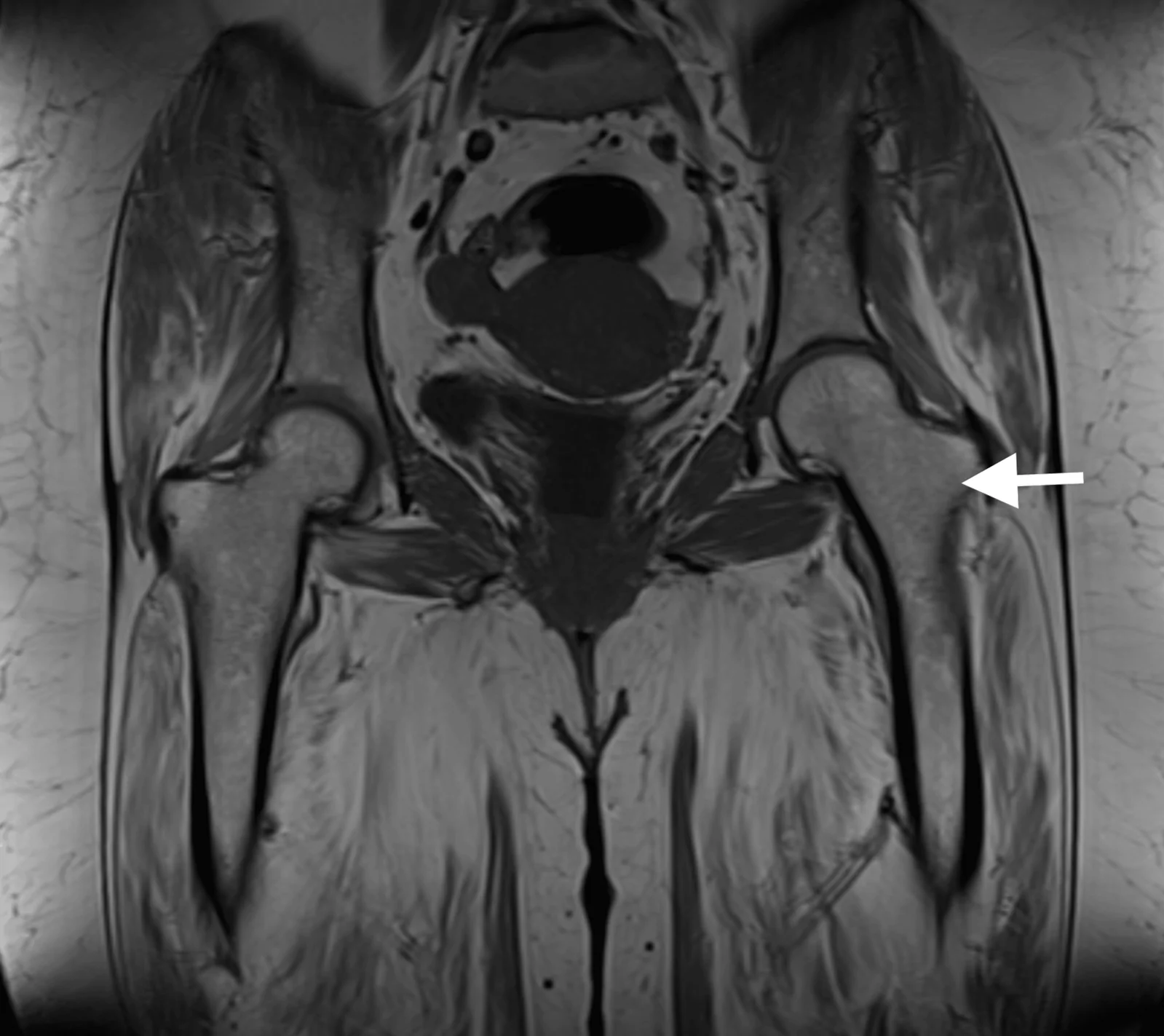
Coronal T1-weighted follow-up MRI of the pelvis showing resolution of the previously observed lesion, with no residual abscess (arrow). The follow-up MRI shows no evidence of residual inflammation or abscess at the previously affected site, as indicated by the arrow. No residual disease is identified.

The aspirated pus tested positive for *Mycobacterium tuberculosis* on nucleic acid amplification testing (Table 2), establishing the diagnosis of tuberculous osteomyelitis with presumed multifocal extrapulmonary involvement. The patient was started on antitubercular therapy. A nine-month antitubercular regimen was used, comprising an intensive phase of two months and a continuation phase of seven months. The drugs used during the intensive phase included isoniazid, rifampicin, pyrazinamide, and ethambutol. Only isoniazid and rifampicin were used during the continuation phase. Ureteric stenting was performed to relieve the obstructive hydronephrosis. The serum creatinine level returned to normal. On follow-up imaging after nine months of antitubercular therapy, MRI showed complete resolution of the proximal femoral and muscular lesions (Figure 7). No enlarged retroperitoneal lymph nodes were observed. Overall, the imaging findings and clinical course supported an excellent response to treatment.

**Table 2:** The table shows a positive nucleic acid amplification test result for Mycobacterium tuberculosis.

Investigation	Result
Specimen	Pus aspirated from the bone abscess
Test	Nucleic acid amplification test
*Mycobacterium tuberculosis* complex	Detected (positive)
Rifampicin resistance	Not detected
Interpretation	Molecular evidence of *M. tuberculosis *infection, consistent with tuberculous osteomyelitis with an intraosseous abscess

## Discussion

This case illustrates the extent to which EPTB can involve multiple anatomic compartments in a single patient and thereby create diagnostic ambiguity. At presentation, the patient had constitutional symptoms, elevated inflammatory markers, and a painful hip, but the disease burden extended beyond the musculoskeletal system to the retroperitoneum and urinary tract. Such a pattern can be mistaken for a multifocal pyogenic infection or malignancy, especially when the initial imaging is evaluated in an organ-by-organ fashion rather than as a unified systemic process [1].

Musculoskeletal tuberculosis most commonly affects the spine; extraspinal disease is less frequent and may be more easily overlooked because clinical findings can be subtle and conventional radiographs may be noncontributory early in the disease course [2-4]. MRI is the modality of choice for evaluating marrow involvement, adjacent soft tissues, joint extension, and abscess formation [3,4]. In the present case, MRI was pivotal in demonstrating the femoral neck/proximal femoral abscess and the associated obturator internus abscess, thereby mapping the full local extent of the disease and indicating a process more complex than isolated septic arthritis or bursitis.

Muscular tuberculosis is rare and may occur via hematogenous dissemination or direct extension from adjacent osseous or nodal disease [4,5]. Involvement of the obturator internus muscle in this patient may have reflected hematogenous dissemination or direct extension from the adjacent proximal femoral lesion. Recognition of this pattern is important because intramuscular tuberculous abscesses can clinically and radiologically mimic pyomyositis or a soft-tissue neoplasm [4,5].

Genitourinary tuberculosis is another classic but underrecognized form of EPTB. Ureteric disease develops from chronic granulomatous inflammation, ulceration, fibrosis, and eventual luminal narrowing, which may culminate in hydroureteronephrosis or hydronephrosis [6-9]. CT is especially useful for delineating hydronephrosis, ureteric wall abnormalities, the site and length of narrowing, associated nodal disease, and other abdominal manifestations [7-9]. In this case, CT clarified the obstructive component by showing inflammatory mid-ureteric narrowing/stricture with left hydronephrosis while simultaneously revealing retroperitoneal lymphadenopathy that supported a disseminated granulomatous process.

The differential diagnosis in this patient included pyogenic osteomyelitis with a secondary soft-tissue abscess, an infiltrative neoplasm with nodal disease, lymphoma, and atypical inflammatory disorders. However, the coexistence of an osseous abscess, muscular involvement, a ureteric inflammatory stricture, and retroperitoneal lymphadenopathy strongly favored a unifying multisystem infection. Microbiologic confirmation, obtained through nucleic acid amplification testing of the aspirated pus, established the diagnosis of tuberculous osteomyelitis with presumed multifocal extrapulmonary involvement. This underscores the importance of image-guided sampling in unusual or multisystem presentations in which imaging alone, although highly suggestive, remains nonspecific [1,3,7].

From a radiologic perspective, the most instructive feature of this case is the complementary use of MRI and CT. MRI was superior for characterizing the proximal femoral and soft-tissue lesions, whereas CT better depicted the nodal disease, obstructive uropathy, and ureteric involvement. Follow-up imaging also served as an objective measure of therapeutic response, documenting regression of the musculoskeletal lesions and resolution of the retroperitoneal and urinary tract abnormalities. Early recognition of EPTB is essential because delayed diagnosis may result in joint destruction, chronic pain, renal compromise, and unnecessary interventions [3,6-9].

Management in such cases is necessarily multidisciplinary. Antitubercular therapy addresses the underlying infection, but adjunctive procedures may be needed when structural complications are present. In this patient, ureteric stenting was an important organ-preserving intervention because imaging showed obstructive uropathy. The favorable interval regression on follow-up MRI and CT illustrates the value of radiologic surveillance, not only for confirming the response but also for ensuring that complications such as a persistent abscess, progressive bone destruction, or unresolved obstruction are not missed.

## Conclusions

Extrapulmonary tuberculosis should remain an important differential diagnosis when imaging reveals multifocal abnormalities involving the bones, muscles, lymph nodes, and genitourinary tract, especially in endemic settings. This case demonstrates how EPTB can present as a radiologic potpourri of seemingly unrelated findings and mimic other infectious or neoplastic entities. Multimodality imaging was central to establishing the extent of the disease, directing microbiologic confirmation through sampling of the bone abscess, and documenting the treatment response. The involvement of the other sites was presumed based on imaging findings and a high index of suspicion in an endemic setting. Prompt diagnosis and treatment can avert both musculoskeletal morbidity and renal complications.
